# The Protective Activity of *Withania somnifera* Against Mercuric Chloride (HgCl_2_)-Induced Renal Toxicity in Male Rats

**DOI:** 10.1155/2024/8023989

**Published:** 2024-10-28

**Authors:** Haddad A. El Rabey, Samar M. Rezk, Aseel Abusaber, Rwaah Khlabi, Ayah H. Alhawiti, Romana M. Algorayed, Nadia Bakry

**Affiliations:** ^1^Biochemistry Department, Faculty of Science, University of Tabuk, Tabuk, Saudi Arabia; ^2^Clinical Nutrition Department, Mahalla Hepatology Teaching Hospital, Ministry of Health and Population, Gharbyia, El-Mahalla El-Kubra, Egypt; ^3^Department of Clinical Pathology, King Khalid Hospital-Tabuk, Ministry of Health, Tabuk, Saudi Arabia; ^4^Department of Clinical Pathology, Bone Marrow Transplantation and Cord Blood Unit, Mansoura University Children Hospital, Mansours, Egypt

**Keywords:** KIM-1, mercuric chloride, oxidative stress, renal toxicity, SDF-1, *Withania somnifera*

## Abstract

The purpose of this study was to test the protective effect of *Withania somnifera* (WS) against the harmful effects of mercuric chloride (HgCl_2_)-induced kidney failure at the histological, biochemical, and immune levels in Wistar rats. The study assessed the biochemical and immunological changes in five groups (*n* = 6): Group 1 (G1) was the negative control, and the other rats received a single subcutaneous dose of HgCl_2_ (2.5 mg/kg in 0.5 mL of 0.9% saline solution) and randomly divided into 4 groups. Group 2 (G2) was the positive control and left without treatment. Groups 3, 4, and 5 (G3, G4, and G5) were treated with different doses of WS root powder for 30 days. The HgCl_2_-positive group showed significant signs of renal toxicity as reflected by increased levels of kidney function parameters (blood urea nitrogen, urea, and creatinine), inflammatory biomarkers, immunological indices (SDF-1, IL-6, NGAL, and KIM-1), and oxidative stress (SOD, TAC, CAT, GSH, and MDA). The positive group rats also showed drastic pathological changes in renal tissues. Different doses of WS treatment significantly reduced the levels of all biochemical markers and decreased pathological damage to the kidney tissues. The antioxidant, phenolic, and flavonoid constituents of WS root powder helped protect rats' kidneys against HgCl_2_-induced kidney toxicity in male rats.

## 1. Introduction

Mercury, a heavy metal, is one of the oldest dangerous industrial and environmental poisons that cause drastic alterations in human and animal body tissues, including the kidney and other organs, and it is considered hazardous or poisonous at low concentrations [1–3]. The kidney is susceptible to drug toxicity because it receives 20%–25% of the heart's resting cardiac output; so, it is more exposed to drugs in circulation than any other organ system; the kidney is exposed to higher drug concentrations because the tubules concentrate the filtrate, drug transporters can further increase intracellular drug concentrations, and it is susceptible to nephrotoxic injury because tubules require a lot of energy which occurs when mercury salts are taken up and stored in the kidneys [1].

When inorganic mercury binds to intracellular carboxyl, sulfhydryl, and phosphoryl groups, it becomes toxic in the kidneys like how it accumulates in proximal tubule epithelial cells [4]. The result of these interactions is enzymatic inactivation, inhibition of protein synthesis [5], inhibition of cellular multiplication, decrease in uridine and thymidine uptake, DNA fragmentation, and cellular death [6]. Because inorganic mercury alters the number of thiols within cells, it can cause oxidative stress, lipid peroxidation, malfunction in the mitochondria, and modifications to heme metabolism [7]. The toxicity of mercury has been the subject of numerous investigations. One of the most dangerous forms of mercury is mostly used by the liver and eventually accumulates in the kidneys. As a result, it is believed that the kidneys and liver are the organs most affected [8]. Superoxide dismutase and catalase are two examples of free radical rummaging frameworks that are crushed by mercuric chloride (HgCl_2_) [9], which also raises the levels of responsive species that aggravate the prooxidant-cell reinforcement balance framework and result in an oxidative pressure state [10].

*Withania somnifera* (WS), commonly referred to as “ashwagandha” or Indian ginseng, is a well-known medicinal herb that has many therapeutic activities in India, particularly in Ayurveda [11, 12]. It is a small, upright evergreen shrub that can reach a height of four to five feet. It has a variety of benefits, including the ability to reverse the effects of heavy metal-induced oxidative stress and to be adaptogenic, anticonvulsant, cytoprotective, anti-inflammatory, immunomodulatory, antiarthritic, and antiapoptotic [11–13]. In addition, it has been shown to have a hepatoprotective effect against the toxicity of paracetamol and acetaminophen [11, 14], with the treatment of liver diseases achieved through inhibition of cyclooxygenase-II, TNF-*α*, interleukin-1-beta (IL-1-*β*), and inducible nitric oxide synthase (iNOS) [15]. Hepatatorenal protection against ND (nandrolone decanoate) toxicity is induced by WS root, and it does so by reversing marker enzymes and biochemical parameters to almost near-normal levels. When WS is administered orally, it protects the kidneys from renal damage caused by gentamicin/cisplatin, bromobenzene, and mitochondrial dysfunction. It also lowers nephrotoxicity and increases levels of liver marker enzymes such as AST, ALT, and ALP as well as kidney markers such as creatinine, uric acid (UA), and blood urea nitrogen (BUN) [16]. Numerous studies have demonstrated that ashwagandha may add about 20% to a person's lifespan when treating cancer, Alzheimer's disease, and other illnesses [17]. WS roots are used in the Middle East and India as in herbal medicine for protecting and ameliorating kidney function [1, 5].

In this study, HgCl_2_-induced toxicity was investigated and the protective benefits of WS root powder against kidney metal-induced toxicity were assessed based on biochemical and histological criteria.

## 2. Materials and Methods

### 2.1. Chemicals

Natural WS (ashwagandha) root powder was purchased from Alharraz commercial company, Cairo, Egypt. HgCl_2_ was supplied by Sigma Chemical Co.

### 2.2. Animals

Thirty Sprague–Dawley strain adult male albino rats weighing 200 ± 5 g were obtained from the Agricultural Research Center located in Giza, Egypt. The animals were housed in stainless steel cages (6/each) in a room with controlled temperature (23 + 2°C) and humidity (60 + 2%) and were kept on a 12-h light-dark cycle (7 a.m.–7 p.m.). Water and basal diet [18] were available *ad libitum* during the experiment, and the laboratory animals' nutritional supply fed the animals a basal diet.

### 2.3. Standard Basal Diet

The basal diet consisted of the following ingredients: casein, corn oil, vitamin mixture, mineral mixture, choline chloride, methionine, cellulose, and corn starch according to AIN-93. While the salt mixture was prepared according to Hegsted et al. [19], the vitamin mixture components employed were prepared according to Campbell [20].

### 2.4. Experiment Design

This work was performed at Mansoura University, Mansoura, Egypt, according to the ethical guidelines of the University of Mansoura. Ethical approval was granted by the Institutional Research Board (IRB) of Mansoura University Animal Care and Use Community (MU-ACUC), Egypt (Key: SC.MS.22.12.4). The rats were randomly assigned to five groups, six rats each, as follows: G1 (the first group: received only one subcutaneous injection dose of 0.5 mL 0.9% saline solution), and the other remaining rats were rats which received a single subcutaneous dose of HgCl_2_ (2.5 mg/kg in 0.5 mL 0.9% saline solution) to induce kidney toxicity [21] and were randomly divided into four groups: an untreated positive control group (G2) and three WS-treated groups. The doses of WS in the three groups were chosen to be around what is used in herbal medicine in the Middle East and India in herbal medicine for protecting and ameliorating kidney function as follows: the third group (G3) received a daily dose of 250 mg/kg of WS root powder using gastric gavage for 30 days, the fourth group (G4) received a daily dose of 500 mg/kg of WS roots powder using gastric gavage for 30 days, and the fifth group (G5) received a daily dose of 750 mg/kg of WS roots powder using gastric gavage for 30 days [22]. The 30 days were found enough to induce kidney toxicity and reveal the protective activity of the treating materials [22].

### 2.5. Euthanasia, Dissection, and Blood Collection

The rats were CO_2_ euthanized in their cages until narcosis and then translocated. Rats were then dissected; blood samples were collected for biochemical analysis, and kidneys were washed in saline and kept in 10% formalin for histological preparations. The blood samples were frozen at −20°C for analysis after being centrifuged at 2000 g for 10 min at 4°C. The samples were then aliquoted for the appropriate analytical findings.

### 2.6. Biochemical Analysis

The following biochemical markers were selected carefully to measure kidney functions, renal toxicity, and the protecting activity of WS.

### 2.7. Kidney Indices

Automated and standardized techniques using a commercial kit (Diamond) from Germany were used to determine serum creatinine. The method used to determine UA was a commercial kit (Diamond). Serum BUN was estimated using a commercial kit (Human). All analyses were done according to the instructions of the suppliers.

### 2.8. Antioxidants

Serum concentrations of reduced glutathione (GSH), malondialdehyde (MDA), hydrogen peroxide (H_2_O_2_), total antioxidant capacity (TAC), and superoxide dismutase (SOD) were measured using the procedures outlined by Chance and Mackley [23], DeChatele et al. [24], Beutler et al. [25], Stocks and Dormandy [26], Aebi [27], and Beutler et al. [25], respectively. In addition, a sandwich solid-phase enzyme-linked immunosorbent assay (ELISA) was used to measure the levels of stromal-derived factor 1 (SDF-1) ng/mL, interleukin-6 (IL-6) (pg/mL), neutrophil gelatinase-associated lipocalin (NGAL), and kidney injury molecule-1 (KIM-1) (pg/mL) using commercial kits (Elabsience, RnD system, Bioassay, and Abcam rat kit, respectively).

### 2.9. Histopathological Preparation

Kidney specimens were obtained, promptly preserved in 10% buffered formalin, dried in an increasing ethanol concentration series (70%, 80%, 90%, and 100%), cleared in xylene, and embedded in paraffin. Hematoxylin and eosin dye were used to generate sections that were 4-5 *μ*m in size [28].

### 2.10. Statistical Analysis

The Statistical Package for Social Science (SPSS, Inc., Chicago, IL, USA) 16.0 Program was used to analyze the results of this study. The expression for data from independent experiments is the arithmetic mean ± standard deviation. One-way analysis of variance (ANOVA) and the Student's *t*-test were used to assess the statistical significance of the mean differences. A significance level of *p* < 0.05 was used.

## 3. Results

### 3.1. Histopathology of the Kidney

**Figure 1:**
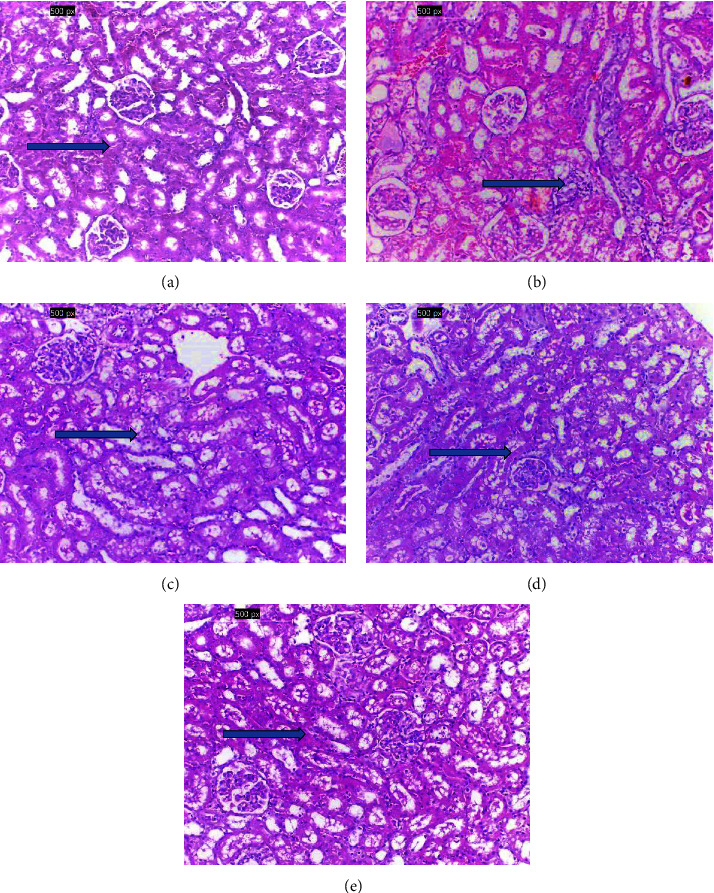
(a) Normal histological structure of renal parenchyma in G1 with normal renal tissue, blood vessels, and interstitial with no histopathological changes (Arrow), (b) marked cortical ischemia with shrinkage of glomerular vascular tuft and marked tubular atrophy (arrow), (c) improved renal tissues (G3) showing moderate tubular atrophy with mild interstitial inflammation (arrow), (d) showing normal glomeruli (G4) with mild tubular atrophy and scantly interstitial inflammation (arrow), and (e) nearly normal renal tissues showing normal cortical tissue with scaled regenerating tubules (arrow) (X200, H&E).

The histological investigations of kidney tissues of various groups are displayed in Figure 1. The control group's renal tissue (G1) showed normal morphological characteristics with normal blood vessels, glomeruli, and interstitial (Figure 1(a)). In contrast, the HgCl_2_-induced kidney toxicity group (G2) exhibited significant renal alteration such as tubular atrophy and cortical ischemia along with glomerular vascular tuft shrinkage (Figure 1(b)). On the other hand, renal tissue treated groups (G3, G4, and G5) showed significant improvement after treatment with WS roots powder at various doses.  Figure 1(c) shows restored and improved renal tissues of G3 with moderate tubular atrophy and mild interstitial inflammation, Figure 1(d) shows normal glomeruli with mild tubular atrophy and scantly interstitial inflammation of G4, and Figure 1(e) shows nearly normal renal tissues with normal cortical tissue and scaled regenerating tubules. The improvement increased with the increase of the treating dose of WS as shown in Figures 1(c), 1(d), and 1(e).

**Figure 2:**
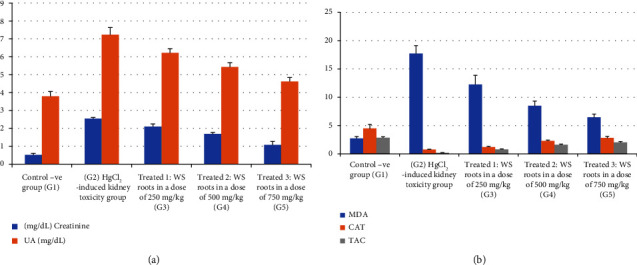
Effect of different doses of *Withania somnifera* on kidney markers, antioxidants, and lipid peroxidation in HgCl_2_-induced kidney toxicity: (a) creatinine and UA) and (b) MDA, CAT, and TAC.

Figure 2(a) and Supporting Table 1 show that the positive control group (G2) had significantly (*p* < 0.05) higher serum creatinine, UA, and BUN in comparison to the negative control group (G1). In contrast to G2, these kidney function parameters were partially restored in the treated groups (G3, 4, and 5). In G5 (treated with WS roots at a dose of 750 mg/kg), the dose was more effective in lowering the kidney function indices, compared with the other treated groups (G3 and G4).

The antioxidants analyses are shown in Supporting Table 2 and Figure 2(b). The activities of SOD and catalase (CAT), as well as the levels of GSH and TAC, were all dramatically reduced as a result of kidney damage induced by the HgCl_2_ injection in the positive control group (G2). In contrast to the positive control group (G2), these markers were elevated in the rats treated with WS roots (G3, G4, and G5).

In addition, Supporting Table 2 also shows that exposure to HgCl_2_ toxicity greatly increased the levels of lipid peroxidation, MDA, and H_2_O_2_ compared with the negative control group (G1). On the other hand, WS treatment in G3, G4 and G5 significantly (*p* < 0.05) decreased MDA and H_2_O_2_ levels compared to that of the positive control. There was a substantial decrease in MDA, a measure of oxidative stress, in G5 (*p* < 0.05) compared with other treated rat groups (groups 3 and 4).

The data presented in Table 1 show that, in comparison with the negative control group (G1), HgCl_2_ toxicity significantly increased the levels of stromal cell SDF-1, KIM-1, NGAL, and IL-6, whereas treatment with WS roots significantly (*p* < 0.05) reduced these elevated parameters in the WS-treated groups (G3, G4, and G5) although the levels remained significantly higher (*p* < 0.05) than those of the negative control group (G1).

On the other hand, Table 2 shows any potential relationships between the parameters under investigation. The study's findings indicate that there were positive and statistically significant connections between the observed antioxidant levels. On the other hand, oxidative stress indicators (H_2_O_2_ and MDA) and measured antioxidants showed substantial negative relationships.

Table 2 shows a positive correlation found between BUN, Il-6, creatinine, and UA. Furthermore, a strong positive correlation was found between the levels of kidney function indices (BUN, UA, and creatinine) and SDF-1 KIM-1, NGAL, and Il-6. Remarkably, Table 2 also shows a strong negative correlation between the measured antioxidants and (NGAL, IL-6, SDF-1, and KIM-1).

## 4. Discussion

The results of this study revealed the toxicity of the male rat's kidney as a result of HgCl_2_ exposure which emphasized that the kidney is sensitive to mercury chloride exposure, which has been identified as a renal toxin [9, 29, 30]. This toxicity occurs because mercury compounds are accumulated in the rats' kidneys [6]. The renal damage was proved by the elevated levels of renal biomarkers, inflammatory markers, immunoglobulin markers, and the drastic renal tissue damage in the kidneys during HgCl_2_-induced kidney toxicity (G2) [6]. The findings of our investigation align with previous publications that indicate a noteworthy rise in BUN, creatinine, and UA levels after exposure to HgCl_2_ [6, 21, 31].

The improvement of renal function indices after treatment with WS root powder is corroborated by earlier research showing that WS roots provided a considerable but partial shield against tubular cell injury and avoided the rise in BUN, UA, and creatinine levels seen in rats given HgCl_2_ in the positive control group [11–14, 31]. The degree of these metabolic profile changes was observed when WS root powder was increased. These results might point to WS root powders' protective mechanism against kidney damage in rats exposed to heavy metals. The assessment of kidney (creatinine, UA, and BUN) indices after WS root powder treatment indicates that WS can reverse the kidney tissue damage produced by HgCl_2_ toxicity; these results are suggestive of the renal protection provided by WS root against nephrotoxicity [31]. The current results emphasized the antioxidant and protective effect of WS powder against HgCl_2_-induced nephrotoxicity as revealed by the improved biochemical analyses is supported by previous investigations [11–13].

The current study's histopathology results showed significant changes to the kidney's architecture caused by HgCl_2_-induced nephrotoxicity [31]. The histopathological evaluation unequivocally shows that the cellular damages in the HgCl_2_-induced nephrotoxicity group, such as necrosis and blood vessel and sinusoidal congestion, may be caused by a reduction in the body's overall antioxidant capacity [6]. In addition, it has been reported that bodybuilders' use of anabolic steroids resulted in a marked increase in the thickness of their renal parenchyma and renal volume, which may indicate kidney dysfunction [32]. Following WS therapy, there was a significant reduction in cellular damages, cytoplasmic alterations, and congestion in all treated groups [11–14].

The current result showed a decrease in antioxidant enzymes (CAT, GSH, and SOD) activity and TAC and an increase in H_2_O_2_ and lipid peroxidation as revealed by the increase in MDA. This may occur thanks to strong thiol-binding agents, mercuric ions, and has been shown to raise intracellular ROS levels and cause oxidative stress, which can lead to tissue damage and nephrotoxicity [31, 33]. This metal's toxicity is linked to the production of superoxide radicals and glutathione depletion [34]. Results from previous studies supported the findings of this study, which show that changes in antioxidant enzyme activities in mercury intoxication are caused by reactive oxygen species (O_2_ or H_2_O_2_), which increase lipid peroxidation levels and decrease glutathione levels [35]. Serum SOD, glutathione peroxidase, and catalase activities, as well as reduced glutathione levels, were also decreased as a result of the induced renal toxicity [33–35].

Supplementing with WS roots may help lessen and moderate the progression of renal disorders, which are otherwise markedly accelerated by oxidative stress. It has already been discovered that dietary WS roots guard against renal toxicity brought on by the negative effects of numerous toxic chemicals [31]. Lipid peroxidation was highly inhibited by WS extracts. Antioxidant activities have been linked to several processes, including scavenging radicals, breaking down peroxides, preventing chain initiation, and reducing capacity [32, 33].

The results showed that the groups treated with WS roots powder restored kidney function in the HgCl_2_-induced toxicity groups. This may suggest a free radical-scavenging mechanism and detoxifying action, which may be connected to the high phenolic and flavonoid contents [11–14]. Anthocyanins, which have been shown to have anti-inflammatory, hydrolytic and oxidative enzyme inhibition, and free radical scavenging capabilities, are the major phenolic compounds in WS [36].

When there are acute or long-term renal injuries, kidney function is typically used to assess the kidney's state by measures such as a detectable drop in urine production, serum level measurements of urea and creatinine, and/or estimation of the BUN to creatinine ratio. There are drawbacks to these tests. For example, even in cases when both kidneys have failed, it takes approximately 24 hours for the creatinine level to increase. Thus, several substitute indicators have been suggested, including SDF-1 [37], KIM-1 [38], and NGAL [39], which are employed as biomarkers of kidney injury and is most substantially upregulated in kidneys injured following ischemia or toxic injury.

The current results are consistent with previous studies that demonstrated nephrotoxicity through increased SDF-1, KIM-1, NGAL, and IL-6 by HgCl_2_ [40]. Liu's study, which examined the nephrotoxicity effect of mercury chloride in mice, found that the compound significantly elevated serum levels of KIM-1 and NGAL as a result of kidney injury, and that HgCl_2_ inhibited the expression of renal transporter that elevated levels of SDF-1 and IL-6 as a result of kidney injury through glomerulosclerosis and albuminuria in mice [41].

The root powder of WS, supported by its numerous bioactive components, is effective in protecting against IL-6, which arises from induced kidney toxicity and leads to an increase in the duration of chronic inflammation [42]. Furthermore, data from earlier studies indicated that therapy with WS significantly inhibited nephritis, proteinuria, TNF-*α*, IL-6, and ROS [43, 44]. These findings are in line with our current investigation, which validates the anti-inflammatory activity of WS by lowering IL-6 and TNF-*α*.

According to data showing a dramatic increase in the production of SDF-1, KIM-1, NGAL, and IL-6, as well as the fact that this increase was significantly inhibited by WS roots powder in all treated groups, our prior findings indicate that enhanced production of ROS through HgCl_2_ amplifies the inflammatory reaction and contributes to the subsequent kidney damage. Accordingly, our data support the notion that oxidative stress and inflammatory indicators are positively correlated [43].

The findings of our investigation about the optimal dosage of WS is from 500 mg/kg to 750 mg/kg on HgCl_2_-induced nephrotoxicity revealed that 750 mg of WS was more effective in shielding the kidney from HgCl_2_-induced nephrotoxicity; however, the precise mechanism responsible for this protection is not known. We think that the low dose of WS concentration; the 250 mg/kg bw is not enough to scavenge free radicals and stop the inflammatory response caused by mercury chloride exposure. In future studies, it is recommended to compare WS groups with other standard chelating agents used in treating mercury toxicity.

## 5. Conclusion

The current findings unequivocally show that HgCl_2_ causes renal toxicity as revealed by the altered biochemical renal markers and the histopathology of the renal tissues, whereas WS has a significant medical benefit and possesses amazing antioxidant characteristics, which may be explained by its ability to scavenge free radicals, boost antioxidant activity when combined with natural antioxidants, and also have immunomodulatory effects. Based on the available research, this plant's powdered root could offer a secure and efficient substitute for traditional medications used in treating renal toxicity disorders.

## Figures and Tables

**Table 1:** Plasma concentrations of stromal cell-derived factor 1 (SDF-1), kidney injury molecule-1 (KIM-1), neutrophil gelatinase-associated lipocalin (NGAL), and IL-6 in control and the different treated groups.

	IL-6 pg/mL	NGAL (ng/mL)	KIM-1 (pg/mL)	SDF-1 (ng/mL)
Control −ve group (G1)	36.32 ± 2.72^#^	1.02 ± 0.29^#^	28.91 ± 3.78^#^	2.24 ± 0.629^#^
HgCl_2_-induced kidney toxicity group (G2)	78.80 ± 3.23⁣^∗^	7.87 ± 0.40⁣^∗^	115.93 ± 7.79⁣^∗^	6.85 ± 0.42⁣^∗^
Treated 1: WS roots in a dose of 250 mg/kg (G3)	61.45 ± 3.42⁣^∗^^#^	6.50 ± 0.23⁣^∗^^#^	93.82 ± 4.48⁣^∗^^#^	5.56 ± 0.25⁣^∗^^#^
Treated 2: WS roots in a dose of 500 mg/kg (G4)	54.95 ± 2.15⁣^∗^^#^	4.88 ± 0.21⁣^∗^^#^	72.98 ± 2.65⁣^∗^^#^	4.21 ± 0.40⁣^∗^^#^
Treated 3: WS roots in a dose of 750 mg/kg (G5)	44.78 ± 1.99⁣^∗^^#^	2.54 ± 0.21⁣^∗^^#^	54.13 ± 3.62⁣^∗^^#^	3.49 ± 0.32⁣^∗^^#^

*Note:* The results are expressed as the M ± SD.

⁣^∗^Shows a statistically significant difference (*p* < 0.05). (^#^) Significant, *p* < 0.05 as compared with the HgCl_2_-induced kidney toxicity group (G2). (⁣^∗^) Significant, *p* < 0.05 as compared with the control −ve group (G1).

**Table 2:** Correlations coefficient (*r*) values in some measured parameters in all groups.

	Creatinine	Uric acid	BUN	IL-6	SDF-1	KIM-1	NGAL	MDA	H_2_O_2_	GSH	CAT	SOD	TAC
Creatinine	—	0.962⁣^∗^	0.952⁣^∗^	0.949⁣^∗^	0.955⁣^∗^	0.969⁣^∗^	0.978⁣^∗^	0.948⁣^∗^	0.920⁣^∗^	−0.967⁣^∗^	−0.943⁣^∗^	−0.906⁣^∗^	−0.965⁣^∗^
UA	0.962⁣^∗^	—	0.943⁣^∗^	0.959⁣^∗^	0.955⁣^∗^	0.975⁣^∗^	0.958⁣^∗^	0.953⁣^∗^	0.931⁣^∗^	−0.969⁣^∗^	−0.925⁣^∗^	−0.890⁣^∗^	−0.962⁣^∗^
BUN	0.952⁣^∗^	0.943⁣^∗^	—	0.960⁣^∗^	0.947⁣^∗^	0.965⁣^∗^	0.955⁣^∗^	0.972⁣^∗^	0.961⁣^∗^	−0.963⁣^∗^	−0.906⁣^∗^	−0.877⁣^∗^	−0.964⁣^∗^
IL-6	0.949⁣^∗^	0.959⁣^∗^	0.960⁣^∗^	—	0.951⁣^∗^	0.962⁣^∗^	0.955⁣^∗^	0.970⁣^∗^	0.960⁣^∗^	−0.967⁣^∗^	−0.895⁣^∗^	−0.876⁣^∗^	−0.957⁣^∗^
SDF-1	0.955⁣^∗^	0.955⁣^∗^	0.947⁣^∗^	0.951⁣^∗^	—	0.962⁣^∗^	0.945⁣^∗^	0.958⁣^∗^	0.925⁣^∗^	−0.959⁣^∗^	−0.931⁣^∗^	−0.904⁣^∗^	−0.952⁣^∗^
KIM-1	0.969⁣^∗^	0.975⁣^∗^	0.965⁣^∗^	0.962⁣^∗^	0.962⁣^∗^	—	0.974⁣^∗^	0.962⁣^∗^	0.943⁣^∗^	−0.977⁣^∗^	−0.940⁣^∗^	−0.905⁣^∗^	−0.976⁣^∗^
NGAL	0.978⁣^∗^	0.958⁣^∗^	0.955⁣^∗^	0.955⁣^∗^	0.945⁣^∗^	0.974⁣^∗^	—	0.949⁣^∗^	0.933⁣^∗^	−0.984⁣^∗^	−0.933⁣^∗^	−0.903⁣^∗^	−0.975⁣^∗^
MDA	0.948⁣^∗^	0.953⁣^∗^	0.972⁣^∗^	0.970⁣^∗^	0.958⁣^∗^	0.962⁣^∗^	0.949⁣^∗^	—	0.956⁣^∗^	−0.960⁣^∗^	−0.910⁣^∗^	−0.880⁣^∗^	−0.966⁣^∗^
H_2_O_2_	0.920⁣^∗^	0.931⁣^∗^	0.961⁣^∗^	0.960⁣^∗^	0.925⁣^∗^	0.943⁣^∗^	0.933⁣^∗^	0.956⁣^∗^	—	−0.945⁣^∗^	−0.870⁣^∗^	−0.876⁣^∗^	−0.943⁣^∗^
GSH	−0.967⁣^∗^	−0.969⁣^∗^	−0.963⁣^∗^	−0.967⁣^∗^	−0.959⁣^∗^	−0.977⁣^∗^	−0.984⁣^∗^	−0.960⁣^∗^	−0.945⁣^∗^	—	0.935⁣^∗^	0.918⁣^∗^	0.979⁣^∗^
CAT	−0.943⁣^∗^	−0.925⁣^∗^	−0.906⁣^∗^	−0.895⁣^∗^	−0.931⁣^∗^	−0.940⁣^∗^	−0.933⁣^∗^	−0.910⁣^∗^	−0.870⁣^∗^	0.935⁣^∗^	—	0.868⁣^∗^	0.955⁣^∗^
SOD	−0.906⁣^∗^	−0.890⁣^∗^	−0.877⁣^∗^	−0.876⁣^∗^	−0.904⁣^∗^	−0.905⁣^∗^	−0.903⁣^∗^	−0.880⁣^∗^	−0.876⁣^∗^	0.918⁣^∗^	0.868⁣^∗^	—	0.889
TAC	−0.965⁣^∗^	−0.962⁣^∗^	−0.964⁣^∗^	−0.957⁣^∗^	−0.952⁣^∗^	−0.976⁣^∗^	−0.975⁣^∗^	−0.966⁣^∗^	−0.943⁣^∗^	0.979⁣^∗^	0.955⁣^∗^	0.889⁣^∗^	—

(⁣^∗^) Significant, *p* > 0.05 in each correlations.

## Data Availability

The data used to support the findings of this study are available from the corresponding author upon reasonable request.

## Nomenclature

AIN-93: Rodent diet
ALP: Alkaline phosphatase
ALT: Alanine aminotransferase
AST: Aspartate aminotransferase
BUN: Blood urea nitrogen
BW: Body weight
ELISA: Enzyme-linked immunosorbent assay
G1: The first group: received only one subcutaneous injection dose of 0.5 mL 0.9% saline solution
G2: Received a single subcutaneous dose of HgCl_2_ (2.5 mg/kg in 0.5 mL 0.9% saline solution) to induce kidney toxicity
G3: Rats received a single subcutaneous dose of HgCl_2_ [21] (2.5 mg/kg in 0.5 mL 0.9% saline solution) to induce kidney toxicity and treated with 250 mg/kg of WS roots for 30 days
G4: Rats received a single subcutaneous dose of HgCl_2_ [21] (2.5 mg/kg in 0.5 mL 0.9% saline solution) to induce kidney toxicity and were treated with 500 mg/kg of WS roots for 30 days
G5: Rats received a single subcutaneous dose of HgCl_2_ [21] (2.5 mg/kg in 0.5 mL 0.9% saline solution) to induce kidney toxicity and were treated with 750 mg/kg of WS roots for 30 days
GSH: Reduced glutathione
IL-6: Interleukin-6
iNOS: Inducible nitric oxide synthase
KIM-1: Kidney injury molecule
MDA: Malondialdehyde
NGAL: Neutrophil gelatinase-associated lipocalin
SDF-1: Stromal cell-derived factor 1
SOD: Superoxide dismutase
SPSS: Statistical Package for Social Science
TAC: Total antioxidant capacity
WS: *Withania somnifera*

## Supporting Information

Supporting Information Supporting Table 1: Plasma concentrations of creatinine, uric acid (UA), and BUN in control and the different treated groups.

Supporting Information Supporting Table 2: Concentrations of oxidative stress parameters and antioxidant biomarkers in control and the different treated groups.
