# Supplementary material for: The Protective Activity of Withania somnifera Against Mercuric Chloride (HgCl2)-Induced Renal Toxicity in Male Rats
Source: Int J Nephrol. 2024 Oct 28;2024:8023989. doi: 10.1155/2024/8023989 (PMC11535192; doi:10.1155/2024/8023989)
Supplement: Supporting Information — Supporting Table 1: Plasma concentrations of creatinine, uric acid (UA), and BUN in control and the different treated groups. [file 8023989.f1.pdf]

Supplementary Table 1: Plasma concentrations of creatinine, uric acid (UA), and BUN in control and the different treated groups

	**BUN (mg/dl)**	**UA (mg/dl)**	**Creatinine (mg/dl)**
Control –ve group (G 1)	18.50 ± 2.32#	3.79± .27#	0.516± .088#
$HgCl_2$ induce kidney toxicity group (G 2)	73.40± 5.79*	7.23± .40*	2.55± .056*
Treated 1 WS roots in a dose of 250 mg/kg (G 3)	53.40± 3.06*#	6.22± .23*#	2.10 ± .14*#
Treated 2 WS roots in a dose of 500 mg/kg (G 4)	41.90± 3.38*#	5.43 ± .23*#	1.68± .087*#
Treated 3 WS roots in a dose of 750 mg/kg (G 5)	32.60 ± 2.45*#	4.62± .21*#	1.07± .198*#

The results are expressed as the M ± SD. * shows a statistically significant difference ($P < 0.05$)

(*) Significant, $p<0.05$; as compared control -ve group (G 1).

(#) Significant, $p< 0.05$ as compared to $HgCl_2$ induce kidney toxicity group (G 2).
