# Supplementary material for: The Protective Activity of Withania somnifera Against Mercuric Chloride (HgCl2)-Induced Renal Toxicity in Male Rats
Source: Int J Nephrol. 2024 Oct 28;2024:8023989. doi: 10.1155/2024/8023989 (PMC11535192; doi:10.1155/2024/8023989)
Supplement: Supporting Information — Supporting Table 2: Concentrations of oxidative stress parameters and antioxidant biomarkers in control and the different treated groups. [file 8023989.f2.pdf]

	**MDA (nmol/ml)**	**H_2O_2 (mM / L)**	**GSH (mmol/L)**	**CAT (U/L)**	**SOD (U/L)**	**TAC (mM/L)**
Control –ve group (G 1)	2.73± .32#	.14± .057#	183.50± 5.79#	4.49 ±.73#	348.90± 67.85#	2.86±.158#
$HgCl_2$ induce kidney toxicity group (G 2)	17.72± 1.37*	1.53 ± .109*	39.80± 4.77*	.76 ±.081*	158.70± 6.48*	0.19±.063*
Treated 1 WS roots in a dose of 250 mg/kg (G 3)	12.27± 1.61*#	.86± .061*#	76.50± 4.17*#	1.22 ±.11*#	208.20± 8.97*#	0.80±.090*#
Treated 2 WS roots in a dose of 500 mg/kg (G 4)	8.51± .81*#	.65± .049*#	112.60 ± 10.28*#	2.30 ± .13*#	243.80±6.05*#	1.62±.128*#
Treated 3 WS roots in a dose of 750 mg/kg (G 5)	6.46± .56*#	.42± .098*#	154.30± 7.98*#	2.84 ±.26*#	311.60±7.60#	2.05±.145*#

Supplementary Table 2: Concentrations of oxidative stress parameters and antioxidant biomarkers in control and the different treated groups

The results are expressed as the M ± SD. * shows a statistically significant difference ($P < 0.05$)

(*) Significant, $p<0.05$; as compared control -ve group (G1).

(#) Significant, $p< 0.05$ as compared to $HgCl_2$ induce kidney toxicity group (G2).
